# 含氟富氮多孔有机聚合物的合成及其对水中全氟辛酸的去除

**DOI:** 10.3724/SP.J.1123.2024.04006

**Published:** 2024-06-08

**Authors:** Xin CHEN, Wenping QIAN, Tianqi CHEN, Lingyun SHAO, Wenfen ZHANG, Shusheng ZHANG

**Affiliations:** 1.郑州大学化学学院, 河南 郑州 450001; 1. College of Chemistry, Zhengzhou University, Zhengzhou 450001, China; 2.郑州大学风味科学研究中心, 中原食品实验室, 河南 郑州 450001; 2. Food Laboratory of Zhongyuan, Flavour Science Research Center of Zhengzhou University, Zhengzhou 450001, China

**Keywords:** 液相色谱-串联质谱, 富氮多孔有机聚合物, 全氟辛酸, 吸附, liquid chromatography-tandem mass spectrometry (LC-MS/MS), nitrogen-rich porous organic polymers, perfluorooctanoic acid (PFOA), adsorption

## Abstract

全氟辛酸(PFOA)在自然环境中难以降解,会通过富集渗透污染水体和土壤,从而对自然环境和人体健康造成影响。开发成本低、效率高、环保的吸附剂实现环境水体中PFOA的高效吸附去除是解决PFOA污染的有效途径之一。本研究采用无溶剂一锅法设计、制备了一种含氟富氮多孔有机聚合物(POP-3F),通过引入氟原子增加了材料的疏水性,增加了主客体分子间的疏水作用、氟-氟相互作用,提升了材料对PFOA的吸附效果。使用扫描电子显微镜(SEM)、傅里叶变换红外光谱(FT-IR)、X-射线衍射仪(XRD)、固体核磁(ssNMR)、X射线光电子能谱仪(XPS)、热分析系统(TGA)等对POP-3F进行了表征。结合液相色谱-串联质谱法(LC-MS/MS),研究了POP-3F在不同pH、盐浓度和腐植酸条件下对PFOA的吸附性能。在pH值为2时,POP-3F对PFOA的去除率最高达到98.6%,可用于去除酸性工业废水中的PFOA。并且POP-3F对于PFOA的去除率几乎不受NaCl和腐植酸浓度的影响,在加入NaCl后,POP-3F表面会形成双电层,可以削弱POP-3F与PFOA之间的静电相互作用,去除率仅下降了1%。腐植酸与PFOA存在竞争吸附,在高浓度腐植酸条件下,POP-3F对PFOA的去除率仅下降了0.73%。在最佳pH条件下考察了吸附等温线和吸附动力学,通过数学模型拟合了实验结果,探究了吸附机理。结果显示,POP-3F的理论容量为191 mg/g,高于活性炭和其他多数吸附剂,表现出较高的吸附容量。此外,POP-3F对PFOA的吸附去除几乎不受基质种类的影响,在模拟自然水中吸附效果略有降低(仅降低0.1%),经过5次吸附-解吸循环后,对PFOA的去除率仅微幅下降(降低0.67%),表明其具有循环使用和可再生性,在实际PFOA污染废水处理中具有广阔的应用前景。

## Abstract (en)

Perfluorooctanoic acid (PFOA) is a persistent contaminant with detrimental effects on the natural environment. This persistence leads to potential enrichment and osmotic transfer, which can affect normal circulation in the environment. PFOA poses significant threats to both the natural environment and human health. Therefore, the development of cost-effective, highly efficient, and environment-friendly PFOA adsorbents is a crucial endeavor.

This paper presents the catalyst-free one-pot synthesis of fluorinated nitrogen-rich porous organic polymers (POP-3F) via a Schiff-base condensation reaction. The reaction between the nitrogen-rich compound 1,4-bis(2,4-diamino-1,3,5-triazine)benzene and *p*-trifluoromethylbenzaldehyde yielded POP-3F. The introduction of fluorine atoms into the nitrogen-rich porous organic polymer enhanced its hydrophobicity, thereby facilitating favorable fluoro-fluorine interactions with PFOA and, thus, improving the efficacy of the adsorbent. Scanning electron microscopy (SEM), Fourier transform infrared (FT-IR) spectroscopy, X-ray diffraction (XRD), solid-state nuclear magnetic resonance (ssNMR) spectroscopy, X-ray photoelectron spectroscopy (XPS), nitrogen adsorption-desorption analysis, and thermogravimetric analysis (TGA) were used to confirm the successful synthesis and characterization of POP-3F.

Liquid chromatography-tandem mass spectrometry (LC-MS/MS) was conducted in negative electrospray ionization (ESI) mode coupled with multi-reaction monitoring mode (MRM). The instrument was equipped with an Atlantis T3 column (100 mm×2.1 mm, 3 μm), and analysis was conducted using an external standard method. The influences of various factors on PFOA adsorption by POP-3F, including pH, salt concentration, and humic acid presence, were investigated. The highest PFOA removal rate (98.6%) was achieved at a pH of 2, indicating the applicability of POP-3F for the effective removal of PFOA from acidic industrial wastewater. The removal rate of PFOA was unaffected by increases in NaCl concentration. This phenomenon can be attributed to electrostatic interactions between the protonated secondary amines in POP-3F and deprotonated PFOA. Upon the addition of NaCl, a double electric layer is formed on the POP-3F surface, with Cl^-^ ions in the outer layer and Na^+^ ions in the inner layer, which weakened these interactions. Humic acid is competitively adsorbed with PFOA. However, POP-3F demonstrated good removal rates even in the presence of high humic acid concentrations in water. Adsorption isotherm and kinetics experiments were conducted at the optimal pH to explore the relevant adsorption mechanism. The results showed a rapid initial adsorption rate, with 95.4% PFOA removal within 5 min. Optimal adsorption equilibrium was achieved within 6 h, and the removal rate decreased by only 0.3% after 24 h. This finding indicates that POP-3F exhibits sustained efficacy for PFOA removal. Langmuir fitting analysis revealed a theoretical maximum adsorption capacity of 191 mg/g for POP-3F; this value surpasses those of activated carbon materials and most other adsorbents, highlighting the superior PFOA-adsorption performance of POP-3F. Additionally, matrix effects minimally affected the removal of PFOA by POP-3F, with only a slight reduction (0.1%) observed in simulated natural water. The recyclability of POP-3F was assessed over five adsorption-desorption cycles. The removal efficenecy exhibited a minor decrease of only 0.67% after five cycles. These results demonstrate the recyclability of the proposed adsorbent, which translates into cost reduction through reusability. This characteristic renders POP-3F a promising candidate for the economical and efficient removal of PFOA from wastewater in practical applications.

全氟辛酸(PFOA)是一种人工合成的典型全氟和多氟烷基物质(PFAS)^[1]^,经常被用作表面活性剂^[2]^在全球范围内大量使用。水体中PFOA来源于氟化工厂、污水处理厂、垃圾填埋场等。PFOA的制造和使用是环境中PFOA的主要来源,氟化工厂未经处理的废水中PFOA的典型浓度为0.34~3.35 mmol/L^[3]^,直接排放会污染环境。而污水处理厂一般没有专门的方法去监测和去除PFOA,导致大多数污水中的PFOA没有得到有效去除而直接排放到水体中,使地下水和土壤受到PFOA的污染,污水处理厂废水中PFOA的浓度几乎高出周围水体的1000倍^[4]^。PFOA不仅能抵抗酸、碱、高温环境,还能在还原剂、氧化剂条件下稳定存在,在自然条件下很难水解、光解和降解^[5,6]^。大量研究^[7,8]^发现,不同地方的水体、土壤、动物组织、植物,甚至空气中均含有PFOA,并且在人的血清和组织中可检测到低水平PFOA(通常为ng/L)^[9]^。PFOA在动物和人体内的含量有着逐步积累的趋势和能力,长期暴露在PFOA污染的环境中会对人们的器官造成影响,例如甲状腺^[10]^、卵巢^[11]^、肝脏^[12]^、脾脏^[13]^等,增加各个年龄段人的患癌风险^[14]^;造成人体的生殖障碍^[15]^,例如引起生殖激素水平紊乱、男性不育、受孕能力下降和儿童性早熟等问题。PFOA在水体中的毒性、危害性和持久性已经引起了全世界的关注。

PFOA可通过氧化法^[16]^、光催化降解法^[17]^、电絮凝法^[18]^、吸附法^[19]^等方法去除,但这些方法成本高、效率低,不适合大范围使用。目前,活性炭^[20]^、离子交换树脂^[21]^、多孔有机聚合物(POPs)^[22]^、共价有机骨架材料(COF)^[23]^等吸附剂都可用于PFOA的吸附去除。多孔有机聚合材料因其具有高的比表面积、可调节的功能位点、质量轻、容量大等优点而受到广泛的关注,可作为去除PFOA的经济、环保、高效的吸附剂材料。

本研究选取1,4-双(2,4-二氨基-1,3,5-三嗪)-苯(BDTB)和对三氟甲基苯甲醛(3F-TMA)作为单体,在无催化剂条件下采用一锅法通过席夫碱反应合成了含氟富氮多孔有机聚合物POP-3F。三氟甲基苯甲醛的引入可提高材料的亲脂性,有助于提升对PFOA的亲和力。结合LC-MS/MS技术进一步研究了POP-3F在不同pH、盐浓度、腐植酸等条件下对PFOA的吸附性能,并探究了POP-3F对PFOA的吸附动力学、吸附热力学、吸附容量和再生性能。结合实验结果解释了其吸附机理,为新材料的开发奠定了理论基础。

## 1 实验部分

### 1.1 实验仪器与试剂

Nexera X2高效液相色谱系统、IR Tracer-100傅里叶变换红外光谱仪(日本岛津公司); SCIEX 6500质谱联用系统(美国AB Sciex公司); Helios G4 CX双束扫描电子显微镜(美国Thermo公司); Empyrean X射线衍射仪(XRD,荷兰Panalytical公司); AVANCE(3)400WB固体核磁(瑞士Bruker公司); AXIS Supra X射线光电子能谱(XPS,英国Kratos公司); ASAP2420-4PM比表面积及孔径分析仪(美国Micro-meritics公司); STA 449 F3热分析系统(德国Netzsch公司); Milli-Q纯水机(美国Millipore公司); DZF-6050真空干燥箱(上海一恒科学仪器有限公司); KSK 2210LHC超声波清洗仪(上海科导超声仪器有限公司); SHZ-D(Ⅲ)循环水式真空泵(巩义市予华仪器有限公司)。

对三氟甲基苯甲醛(98%)、*N*,*N*-二甲基甲酰胺(DMF, AR)、氯化钠(99.8%)、腐植酸(≥90%)均购自上海麦克林生化科技有限公司;甲醇(AR)、二甲基亚砜(DMSO, AR)均购自国药集团化学试剂有限公司;PFOA(98%)购自上海迈瑞尔化学技术有限公司;色谱纯甲醇购自美国默克公司。

### 1.2 材料制备

BDTB合成方法参考课题组之前的文献^[24]^, POP-3F根据文献[25]的方法稍作修改。

将BDTB(1185.2 mg, 4 mmol)、3F-TMA(1393 mg, 8 mmol)和DMSO(60 mL)置于100 mL双颈圆底烧瓶中混匀。在氮气气氛下180 ℃加热反应24 h,将产物用10 mL DMSO和甲醇在10000 r/min条件下各离心洗涤3次,用甲醇索氏提取24 h后在120 ℃下真空干燥,得到的POP-3F为凝胶状固体,研磨后为白色粉末,收率为40.22%。POP-3F的合成路线见图1。

**图 1:**
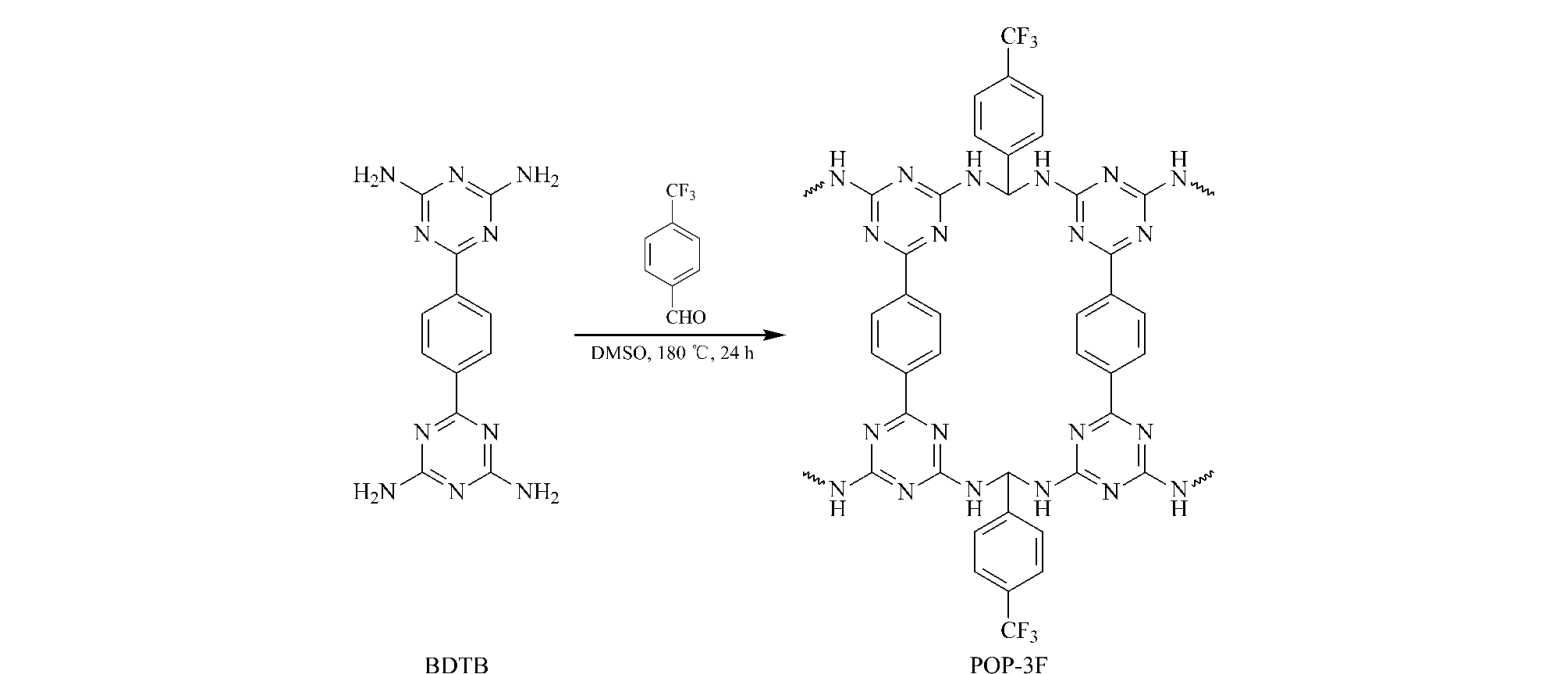
POP-3F的合成示意图

### 1.3 LC-MS/MS方法

Atlantis T3色谱柱(100 mm×2.1 mm, 3 μm,美国Waters公司);流动相5 mmol/L乙酸铵(A)和甲醇(B);柱温40 ℃;流速0.2 mL/min,进样量2 μL。梯度洗脱程序:0~14 min, 80%A~10%A; 14~16 min, 10%A; 16~16.01 min, 10%A~80%A; 16.01~20 min, 80%A。

电喷雾电离(ESI),负离子模式;多反应监测模式(MRM);离子源温度:500 ℃;离子源电压:-4500 V;气帘气压力:2.41×10^5^ Pa;雾化气压力:2.76×10^5^ Pa;辅助器压力:2.76×10^5^ Pa。其他质谱参数见表1。

**表 1:** PFOA的质谱参数

Compound	Parent ion (m/z)	Daughter ions (m/z)	Collision energies/eV
PFOA	413	369^*^/169	-14/-24

* Quantitative ion.

### 1.4 PFOA标准曲线绘制

PFOA的定量采用外标法,首先用去离子水配制质量浓度为100 mg/L的PFOA储备液,再用去离子水稀释为100、50、10、5、1、0.1 μg/L的标准工作液。用LC-MS/MS分析上述标准工作液,以PFOA的质量浓度为横坐标(*x*, mg/L),峰面积为纵坐标(*y*),绘制标准曲线。

在最优条件下,PFOA在0.1~100 μg/L范围内线性关系良好,回归方程为*y*=2.04×10^6^*x*-1.13×10^6^,相关系数(*r*^2^)为0.999。方法的检出限(LOD, *S/N*=3)为0.004 μg/L,定量限(LOQ, *S/N*=10)为0.013 μg/L。

### 1.5 吸附实验

取50 mL 1 mg/L的PFOA溶液,将溶液pH调节至2,再加入10 mg POP-3F,超声1 min使POP-3F固体分散开。然后在25 ℃下以200 r/min恒温振荡吸附24 h,吸附后经过滤将POP-3F与上清液分开,得到的上清液经聚醚砜针式过滤器(0.22 μm×13 mm)过滤后进行LC-MS/MS分析。吸附实验所需器具均由聚丙烯(PP)材质制成,整个过程避免接触聚四氟乙烯和玻璃材质的物品。

### 1.6 脱附实验

根据参考文献[26,27],选择甲醇为洗脱剂进行脱附实验,稀释储备液配制质量浓度为1 mg/L的PFOA溶液(pH=2),再加入10 mg的POP-3F超声1 min。在25 ℃下以200 r/min恒温振荡6 h后通过0.2 μm的针式过滤器(聚醚砜膜)过滤,将所得固体分散在50 mL甲醇中,超声30 min,过滤后在24 h内进行LC-MS/MS分析。

### 1.7 材料的吸附性能

#### 1.7.1 吸附动力学

采用1.5节的方法进行吸附实验,在振荡间隔时间为5、10、20、30、60、120、240、360、720、1440 min时分别用注射器取300 μL的溶液,用LC-MS/MS测定。*t*时间下的吸附量(*q_t_*, mg/g)和去除率(*R*)的计算公式如下:


(1) *q_t_*=(*C*_0_-*C_t_*)*V/m*

(2) *R*=(*C*_0_-*C_t_*)/*C*_0_×100%

式中,*C*_0_和*C_t_*分别表示吸附前和*t*时间时溶液中PFOA的质量浓度(mg/L); *V*表示溶液的总体积(L); *m*表示吸附剂的质量(g)。

#### 1.7.2 吸附等温线

取50 mL一定浓度(1、3、5、7、9、12、15、20 mg/L)的PFOA溶液,采用1.5节的方法进行吸附实验,并根据式(3)计算平衡吸附量*q*_e_(mg/g)。


(3) *q*_e_=(*C*_0_-*C*_e_)*V/m*

式中,*C*_e_表示吸附平衡时溶液中PFOA的含量(mg/L)。

## 2 结果与讨论

### 2.1 POP-3F的表征

通过红外光谱对POP-3F材料进行表征,图2a中,BDTB在大于3200 cm^-1^处的N-H特征峰和3F-TMA在2951 cm^-1^处的-CO-H以及1699 cm^-1^处的C=O特征峰几乎消失。POP-3F在1543 cm^-1^处出现了尖锐的三嗪环特征峰,在3406 cm^-1^处存在宽的C-NH-C吸收峰,并且在1620 cm^-1^处不存在典型的C=N特征峰,表明BDTB和3F-TMA中发生了席夫碱缩合反应,得到了C-NH-C键连接的POP-3F。

**图 2:**
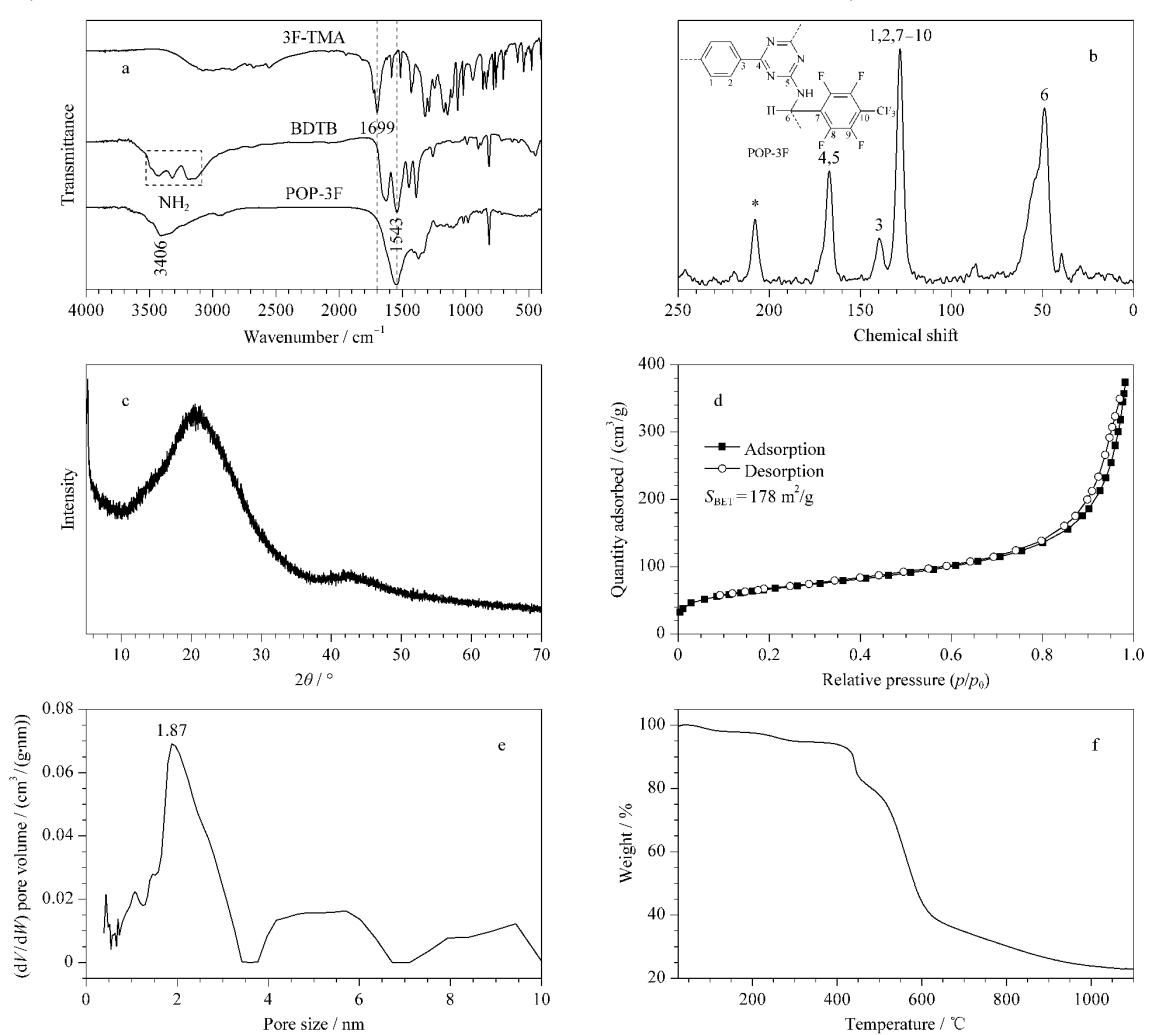
POP-3F的表征图

图2b是POP-3F的^13^C ssNMR谱图。图中化学位移为48.88的峰归属于POP-3F中的烷基碳原子,化学位移为139.94和128.17的峰归属于POP-3F中的苯环碳原子,化学位移为167.00的峰归属于POP-3F中的三嗪环碳原子,在化学位移为207.75的峰归属于未反应的3F-TMA中的醛基。通过^13^C ssNMR谱图再次证明了BDTB和3F-TMA发生缩合反应后生成了C-NH-C键连接的POP-3F。

图2c显示了POP-3F的XRD图,POP-3F仅在2*θ*=20.70°存在一个宽峰,表明POP-3F是无定形结构,这与席夫碱缩合的随机无序性相对应。图2d显示了在77 K的测试条件下测得的POP-3F氮气吸附-脱附等温线图,结果显示吸附和脱附曲线几乎重合,是典型的Ⅱ型吸附等温线特征。基于Brunaner-Emmet-Teller(BET)模型,得到POP-3F的比表面积为178 m^2^/g。在图2e中,可以看出POP-3F中孔径分布主要存在1.87 nm的微孔和少量的介孔(孔径由NLDFT/GCMC方法计算)。在27~1100 ℃范围内考察了POP-3F材料的热稳定性,由图2f可以看出,POP-3F在27~420 ℃范围内重量下降8%,曲线较平缓,是因为材料中水分蒸发和吸附气体的脱附。POP-3F在420 ℃开始分解,导致重量急剧下降,当温度升至620 ℃时,下降速率变得减缓,最终残留23%的重量。结果表明POP-3F具有极高的热稳定性,可应用于实际环境样品的吸附处理。

用扫描电子显微镜对合成的POP-3F材料进行微观形貌表征,图3显示材料的SEM图像是小颗粒块体、微米级结构。

**图 3:**
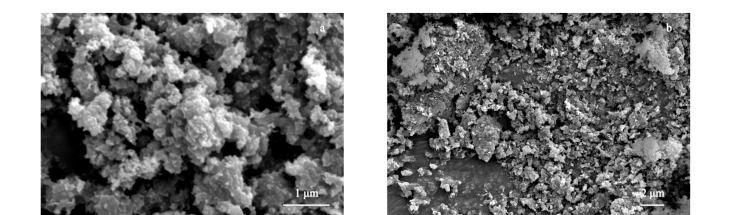
POP-3F的扫描电镜图

对POP-3F进行了XPS表征,观察其原子化学环境,在图4中可观察到C 1*s*、N 1*s*、O 1*s*和F 1*s*的存在。C 1*s*光谱的高分辨率XPS光谱可被分解为284.6、287.2和291.7 eV的3个峰,分别归属于C-C/C-H、三嗪环碳和三氟甲基碳。N 1*s*光谱的高分辨率XPS光谱可在398.3和399.4 eV处被分解为两个峰,分别被归属于三嗪和仲胺的氮原子上。在F 1*s*光谱中只有一个峰,证明POP-3F中只有一种氟原子。POP-3F因为吸附有少量水分,会存在少量的氧元素。

**图 4:**
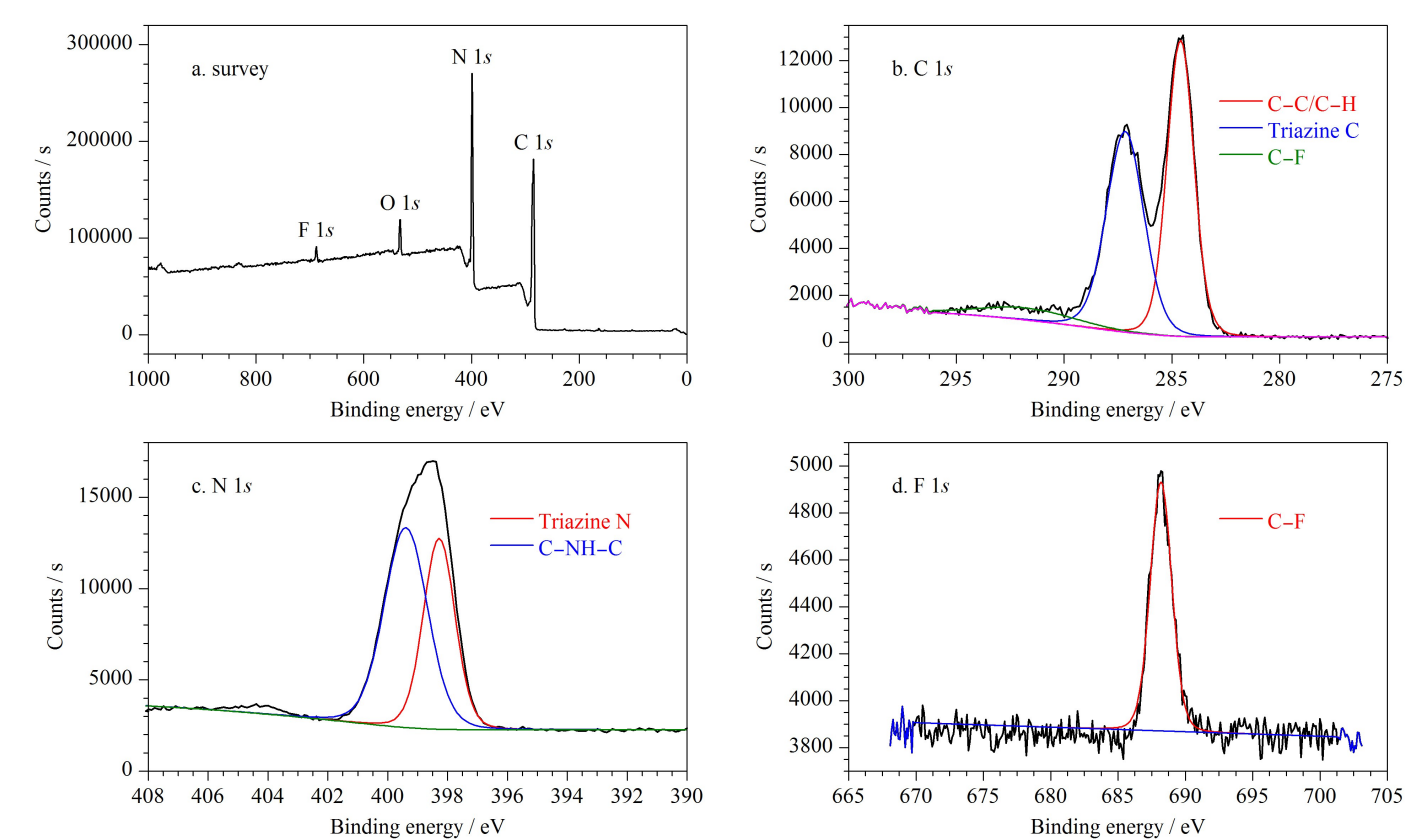
POP-3F的X射线光电子能谱

POP-3F的XPS定量分析表明其元素含量分别为64.57%(C)、31.60%(N)、3.12%(O)、0.72%(F)。XPS所测得的F元素含量与理论值相差较大,是因为XPS分析深度约为2 nm,分析结果为材料表面的元素含量。

### 2.2 吸附性能的优化

#### 2.2.1 吸附动力学

为了考察POP-3F对PFOA的吸附平衡时间,研究了POP-3F对PFOA的吸附动力学,由图5a结果可知,5 min内POP-3F对PFOA的去除率高达95.4%,当吸附时间升至6 h时,吸附平衡达到最佳效果,此时去除率为99.3%。在6 h后,随着吸附时间继续增加,会出现缓慢解吸导致去除率略微下降,但总体解吸程度较低,与6 h吸附平衡时的去除率相比,在24 h时去除率仅下降了0.3%,表明POP-3F是去除PFOA的较好材料。

**图 5:**
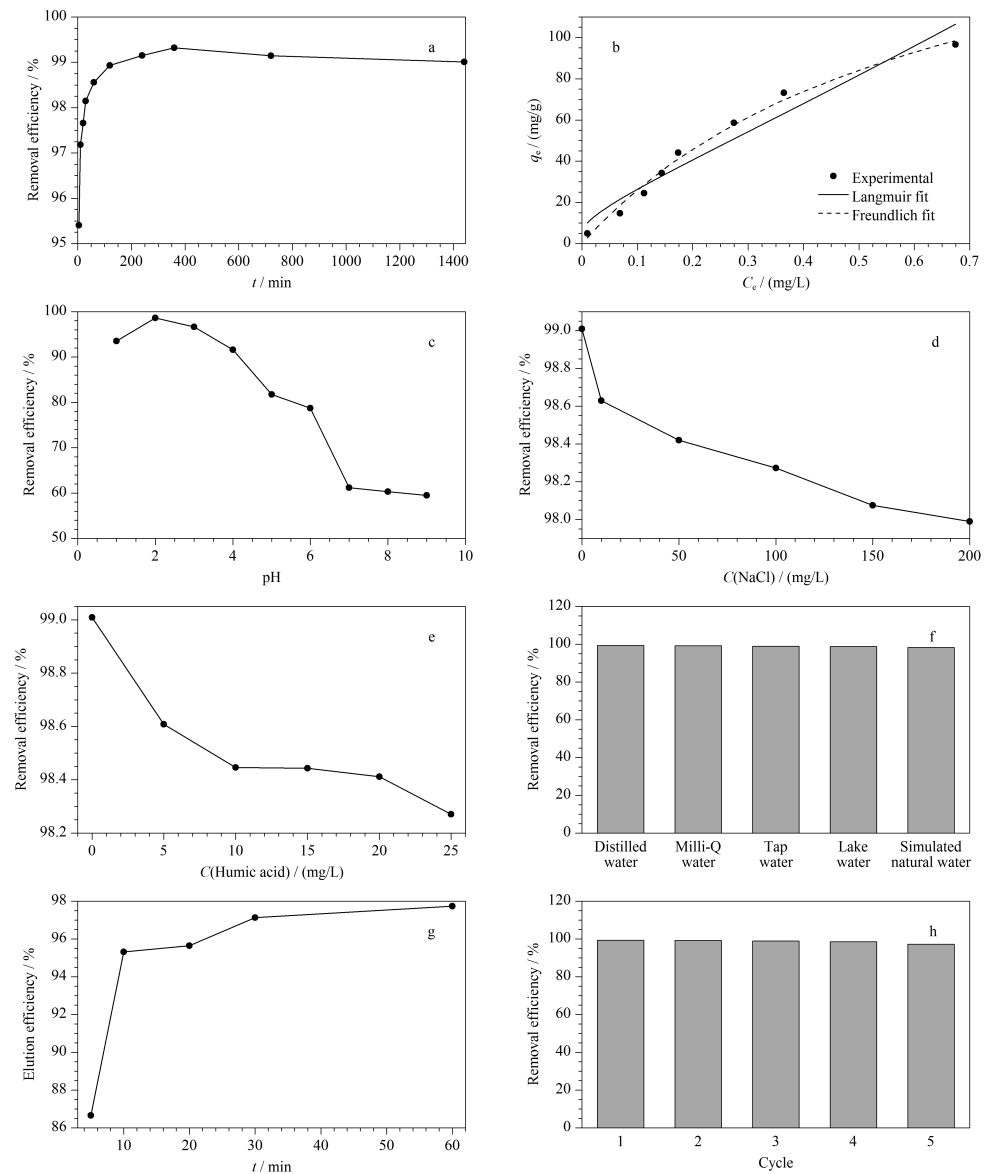
POP-3F的吸附性能

#### 2.2.2 吸附等温线

为了更充分地了解PFOA的吸附机理,评估材料的吸附能力,在298 K对POP-3F进行等温吸附实验,所得数据用Langmuir和Freundlich等温线模型拟合。图5b中,Langmuir模型与实验数据非常吻合(*R*^2^为0.99),说明POP-3F对PFOA的吸附过程更符合Langmuir吸附模型,在其吸附过程中单层吸附占主导作用。根据Langmuir模型拟合得到POP-3F对PFOA的最大吸附理论容量为191 mg/g,这一数值高于活性炭材料^[28,29]^和其他吸附剂^[30]^,说明POP-3F在实际应用中可用于处理PFOA污染的废水。

#### 2.2.3 pH的影响

通过调节水样的pH(1、2、3、4、5、6、7、8、9),考察了pH对去除率的影响。图5c研究了POP-3F的最佳吸附pH值,可观察到在pH等于2时,POP-3F对PFOA的去除率最高,为98.6%; pH为1时,相比于pH为2时的去除率下降了5.1%,为93.5%;当pH在4~9时,POP-3F对PFOA的去除率逐渐下降,在pH等于9时去除率显著下降到59.5%。pH的变化会引起POP-3F中的仲胺质子化程度的变化,随着POP-3F中仲胺质子化程度增大,当PFOA也处于去质子化状态时,主客体分子之间静电作用最强^[31]^。pH等于2时,POP-3F中的仲胺质子化程度较大;pH小于2时,PFOA去质子化稍有减弱;pH大于4时,POP-3F质子化明显减弱,这会降低主客体分子之间的静电作用,导致去除率明显下降,因此POP-3F与PFOA之间的静电作用主导了吸附过程,与文献[32]报道一致。结果表明,POP-3F对去除酸性工业废水中的PFOA有很大的潜力。

#### 2.2.4 盐浓度的影响

为了考察盐浓度对去除率的影响,调节水样中NaCl的质量浓度(200、150、100、50、10 mg/L)。从图5d中可以看出,随着NaCl质量浓度从0增加到200 mg/L, POP-3F对PFOA的去除率逐渐下降。这是因为POP-3F中的仲胺质子化与PFOA的去质子化会发生静电作用,而NaCl的加入会在POP-3F表面形成Cl^-^和Na^+^双电层(Cl^-^在外层,Na^+^在内层),从而减弱了它们之间的静电相互作用。随着NaCl质量浓度的增加,POP-3F表面正电性逐渐减弱,使其去除PFOA的速率逐渐降低,但总体去除率仅下降了1%,说明POP-3F具有较高的抵御NaCl影响的能力,从而有实际应用的潜力。

#### 2.2.5 腐植酸的影响

腐植酸中的酸性物质会与PFOA发生竞争吸附,从而影响POP-3F的去除率,为了验证不同质量浓度的腐植酸对POP-3F去除率的影响,进行了吸附实验。

结果如图5e所示,腐植酸的质量浓度为0~5 mg/L时,POP-3F对PFOA的去除率下降相对明显(下降了0.4%)。腐植酸质量浓度大于5 mg/L后,随着质量浓度的增加去除率缓慢下降。当腐植酸质量浓度为25 mg/L时,去除率为98.27%,比不加腐植酸去除率下降了0.73%。实验结果表明POP-3F在含有高浓度腐植酸条件下仍对水中PFOA有很好的去除效果。

#### 2.2.6 基质影响

水体中有很多的物质可能会影响吸附剂在实际情况中的使用,为了探究POP-3F在复杂水质中的应用能力,选用蒸馏水、Milli-Q水、自来水、湖水、模拟自然水5种水质来观察水体基质对POP-3F去除PFOA效果的影响。从图5f中可以看出,在不同水质条件下,蒸馏水和Milli-Q水中吸附效果最佳,在模拟自然水中吸附效果略有降低(仅降低0.1%),可见水体中的基质种类对POP-3F的吸附性能影响较小。

#### 2.2.7 脱附时间优化

脱附时间会影响POP-3F的脱附效果,实验考察了脱附时间分别为5、10、20、30、60 min时的脱附效果。实验结果如图5g所示,脱附5 min后,PFOA即可洗脱86.6%;当脱附时间从5 min增加到30 min时,洗脱效率明显提高,在30 min时几乎洗脱完全(97.1%)。再增加脱附时间至60 min时,洗脱效率为97.7%且无明显增加。因此选用脱附时间30 min作为POP-3F脱附PFOA的最佳条件。脱附实验过程中使用有机膜(尼龙,0.2 μm×50 mm)过滤得到固体吸附剂。

#### 2.2.8 吸附剂再生实验

材料能否循环利用是体现绿色化学的一个重要方面,以甲醇作为脱附溶剂,对POP-3F材料进行吸附-脱附实验。在图5h中可以看出,在经过5次吸附-脱附循环后,POP-3F对PFOA的去除率从第1次的99.86%下降到第5次的99.19%,去除率没有明显下降。POP-3F能够循环使用,其可再生性可进一步降低成本,说明具有较高的实际使用价值。

## 3 结论

本文通过无溶剂一锅法成功合成了一种含氟富氮多孔有机聚合物POP-3F,在POP-3F中引入三氟甲基可有效提高材料与PFOA之间的静电相互作用和氟-氟相互作用,进而提高POP-3F对PFOA的吸附亲和力。利用LC-MS/MS进行吸附实验,发现在酸、盐和腐植酸存在的情况下,POP-3F对PFOA仍有很好的去除效果,且具有良好的可循环使用性能。本文提出的POP-3F材料合成过程简单,具有作为经济、环保、高效的PFOA吸附剂的潜力。
